# Forecasting Day-Ahead Electricity Metrics with Artificial Neural Networks

**DOI:** 10.3390/s22031051

**Published:** 2022-01-28

**Authors:** Milutin Pavićević, Tomo Popović

**Affiliations:** Faculty of Information Systems and Technologies, University of Donja Gorica, 81000 Podgorica, Montenegro; tomo.popovic@udg.edu.me

**Keywords:** artificial neural networks, day-ahead electricity price forecasting, price forecasting, electricity load forecasting, convolutional neural networks, recurrent neural networks, temporal convolutional neural networks

## Abstract

As artificial neural network architectures grow increasingly more efficient in time-series prediction tasks, their use for day-ahead electricity price and demand prediction, a task with very specific rules and highly volatile dataset values, grows more attractive. Without a standardized way to compare the efficiency of algorithms and methods for forecasting electricity metrics, it is hard to have a good sense of the strengths and weaknesses of each approach. In this paper, we create models in several neural network architectures for predicting the electricity price on the HUPX market and electricity load in Montenegro and compare them to multiple neural network models on the same basis (using the same dataset and metrics). The results show the promising efficiency of neural networks in general for the task of short-term prediction in the field, with methods combining fully connected layers and recurrent neural or temporal convolutional layers performing the best. The feature extraction power of convolutional layers shows very promising results and recommends the further exploration of temporal convolutional networks in the field.

## 1. Introduction

Compared to other commodities traded on free markets under spot and derivative contracts, electricity is very special, as the demand and supply of this economically non-storable commodity must meet at all times. This leads to electricity market price dynamics not observable elsewhere, and characterised by peculiar seasonality and high price volatility [1].

This paper is an extension of the paper “Forecasting Day-Ahead Electricity Price with Artificial Neural Networks: a Comparison of Architectures” [2] by the same authors and expands the research by adding electricity load prediction and comparing the ANN architectures on this additional task.

From the mid 2000s, the way electricity is produced in Europe started changing. Feed-in tariffs, renewable energy subsidies, and tariffs on CO_2_ emissions have made the way this market operates more complex. In addition, the resulting increase of renewable sources which depend on natural factors, such as insolation, wind speed, and time of the year, has made the production more stochastic and increased the volatility of the market prices.

The demand for the day-ahead forecasting of the demand and price of electricity is growing, and using artificial neural networks (ANN) for the task is increasingly explored. The rise of LSTM (long short-term memory) RNN (recurrent neural network) architectures in 2009 [3] made them a go-to choice for this task. Lately, temporal convolutional neural networks (CNNs) have proven to be even more precise on a subset of tasks [4].

There are various approaches to predicting the power market metrics and they can be divided into six classes [5]:

- Production cost models, which simulate agents operating in the market, with the goal to satisfy their demands at minimum cost.
- Game theory approaches, creating equilibrium models to build the price processes.
- Fundamental methods, which map the important physical and economic factors and their influence on the price of electricity market metrics.
- Econometric models, which work with statistical properties of the market metrics over time, to help make decisions in risk management and derivatives evaluation.
- Statistical approaches, implementing statistical and econometric models for forecasting (e.g., similar day, exponential smoothing…)
- Artificial intelligence techniques, which create non-parametric models based on computational intelligence such as fuzzy logic, support vector machines or artificial neural networks.

Artificial neural networks, with their straightforward implementation and promising performance for short-term prediction in the field are probably the most popular AI technique [5,6] explored in the field. They are characterized by their ability to handle complexity and nonlinearity, and ability to adapt to complex and dynamic systems. However, with the ever-growing number of architectures and due to their non-linearity and multi-parameter specifications, comparing these approaches and choosing the optimal one is very hard [6].

Analysing publication trends, R. Weron notes that the electricity price forecasting has saturated the research community, but that the small number of books and review articles on this topic show that the area has yet to reach its maturity [6]. He also notes that there is no industry standard for comparing the efficiency of the prediction algorithms and models, and that error benchmarks used in literature vary a lot, making multi-paper comparisons such as that undertaken by Aggarwal et al. [5,7] something to be treated with caution. Comparing prediction methods, models, and approaches is hard, as they rarely share the same datasets or prediction windows.

With this in mind, we see value in comparing various algorithms on the same ground, and in this paper, we test the performance of various ANN architectures on identical datasets and compare them using the same benchmarks, to gain insights concerning how they perform when predicting hourly day-ahead electricity price and load. These architectures were optimized to give the best performance on their own, but they have not been given any optimization outside neural network model. We aim to create a fair comparison of multiple ANN approaches, with the goal to make the results a usable starting ground for those creating more complex prediction software using ANN architectures.

In addition to traditional neural networks and recurrent neural networks with LSTM cells, often used for this type of prediction, we have also created and tested the performance of temporal convolutional neural (TCN) models, as a relatively new approach to time-series prediction that has shown promising results for certain tasks such as weather forecasting [8]. Comparing TCN results on with RNN/LSTM models in this way provides an insight concerning if (and when) they should be further explored when predicting short-term electricity metrics.

We use the Hungarian HUPX market day-ahead price dataset [9] for the price prediction, and the Montenegrin dataset [10] for load prediction. In addition to ANN models, and in order to evaluate the value of using ANNs in the first place, we have created naive and linear regression models for the same dataset and compared them using identical benchmarks. When predicting electricity load, we also compare the models to the official prediction, supplied by the ENTSO-E transparency platform.

## 2. Materials and Methods

### 2.1. Datasets

A dataset of hourly prices (in EUR, Table 1) for the HUPX day-ahead market was used. In the interests of better data windowing, two non-24 h for every year (due to daylight savings time the last Sunday of October lasts 25, and the last Sunday of March lasts 23 h) were reported as 24 h by the HUPX: the price for the missing hour was reported as zero, and the price for the doubled hour was removed from the dataset. Thus, it consisted of a total of 82,823 hourly values, representing 3451 days between 10 July 2010 and 31 December 2019.

A land area averaged dataset of hourly weather data for the Hungary was provided by the Weather Ninja service and coupled with the market data. As HUPX does not cover only the Hungarian market and the weather was not the only factor influencing the price, this brief dataset was far from complete and should be expanded for use in final products. However, it provided a field for the comparison of architectures.

The dataset was expanded with derived data, as month, day of month, and weekday values were separately added. As the aim was to compare the general efficiency, and not to maximize the precision by further expert input, no data on holidays or similar expectation markers, such as negative price or power facility maintenance expectation, were added.

Furthermore, a separate test was conducted on a dataset expanded with naive seasonal data calculation, namely the daily price difference between the prediction hour and the same hour on the same date a year ago. This dataset was somewhat shorter, containing values from 1 January 2011, with EPEX data being used for calculations between 1 January and 10 July 2010 as the German market was used in place of HUPX prior to its formation. These values introduced serious overfitting issues to the training process, and their usefulness was defined by how well the model can be optimized to avoid overfitting.

To prepare the dataset for ANN prediction, the data frame was divided into three sets, namely training, validation, and testing, in the proportion of 70, 30, and 10, respectively. The training set contained the data used for creating the model, the validation set was used for evaluation during training, and the test set was set aside, to test and evaluate models once they were created.

In addition, the classification information (day, week, and month columns) was turned into one-hot arrays. The rest of the columns were standardized (normalized) into Z-values (standard score).

**Figure 1:**
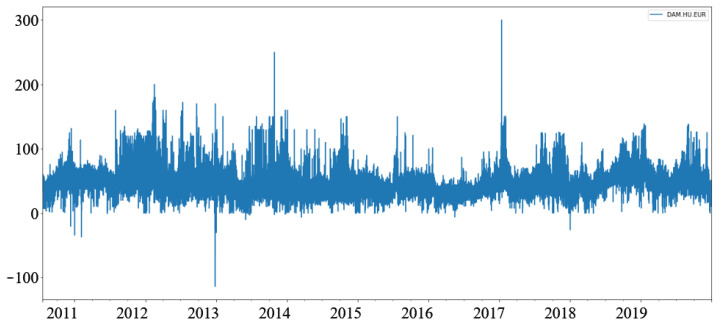
Hourly electricity prices on day ahead HUPX market.

**Figure 2:**
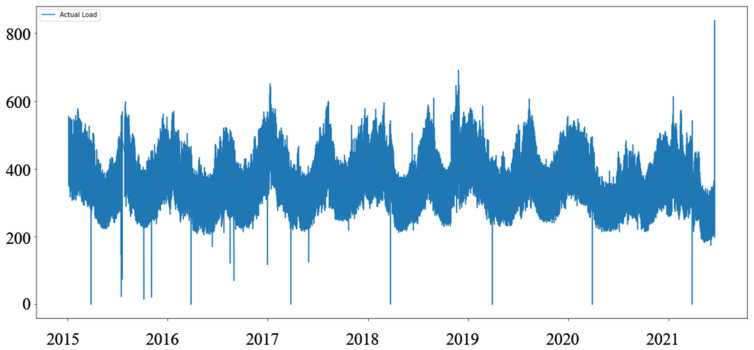
Hourly electricity consumption (load) for Montenegro.

The dataset for the day ahead load (power consumption, Table 2) for Montenegro is taken from the ENTSO-e transparency [10], and covers the period between 1 June 2015 and 19 June 2021. Unlike the price dataset, the weather data were not present, but a column with a one-hot value describing holidays was added. Comparing the two datasets (Figure 1 and Figure 2), we notice that the load data are less stochastic than the price data.

As ENTSO-e data for Montenegro shows two non-24-h days every year. Due to daylight savings time (visible as dips to zero in Figure 2), the dataset was modified to allow for the better fitting of prediction windows. The extra hour in 25-h days was deleted, and the missing hour in the 23-h days was added as a clone of the previous hour.

In addition, a column with official forecast load published by the Montenegrin operator was added. This value was not used for prediction in any way and was used only for comparison purposes. A total of 56,394 rows were present, with columns listing actual load, forecasted load, and one-hot values representing hour, month, weekday, and a holiday marker (1 if the next day is holiday, 0 if it is not). Values that are not one-hot were standardized into Z-values.

It is worth noting that models based on two datasets predict different measures—prices in EUR (Table 1, Figure 1) and Load in MW (Table 2, Figure 2). Exploring these datasets, we see daily, weekly, and annual seasonality, but the price dataset features prominent sudden spikes that are short-lived [6], making prediction harder.

### 2.2. Software and Data Windowing

The code was written in Python, with Keras [11] and TensorFlow [12] libraries used to build machine learning models, Pandas [13] for data manipulation, and Numpy [14] for array and matrices support.

ANN models and data windows were built following the methods described by J. Brownlee [15], adapted and expanded for the task at hand. However, the language used to describe some concepts such as “multi-layer perceptrons” differs, in accordance with the advice given by M. Nielsen [16].

**Figure 3:**
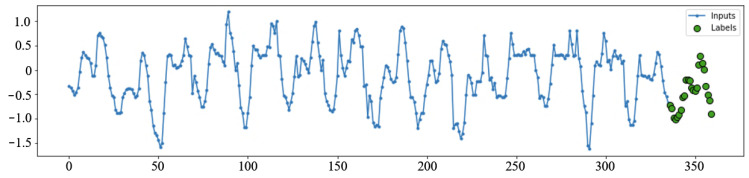
Example data windows with inputs and labels.

The ANN models are given data windows of 14 days of consecutive hourly values (inputs) and are asked to perform prediction for the next day (24 hourly labels, Figure 3).

The task given to the ANN models for price and load prediction can be described as follows:

- Given the total of 336 hourly day-ahead prices on the HUPX market for 14 consecutive days, forecast the 24 day-ahead hourly prices for the next day.
- Given the total of 336 hourly load values for 14 consecutive days, forecast the load for 24 h of the next day.

Every window in the training dataset starts at the same hour. Every day in the dataset is, therefore, once in the labels position and 14 times in the position of inputs (once for each of the 14 positions).

The price prediction models took 8–24 epochs to train. The load prediction models took 14–35 epoch to train, indicating that their gradient is somewhat more fit for machine learning.

### 2.3. Objective Function, Evaluation and Comparison

The objective function (loss) used in neural network training defines how the neural network treats the errors and estimates their severity. Due to the differences in datasets, and even between time periods within the same dataset, a common way of reporting the accuracy is mean absolute percentage error (MAPE) [17,18], i.e., the mean value, in percent, of an absolute value of forecast error divided by the actual value.


(1) $$\mathrm{MAPE}(w,b)=\frac{100}{n}\sum_{i=1}^{n}\left|\frac{y\left(x_{i}\right)-a_{i}}{y\left(x_{i}\right)}\right|\%$$

MAPE, however, shows weaknesses with datasets like the one at hand, where labels with zero or close-to-zero values are present [19], which is especially the case for electricity prices. Even with measures put in place to minimize exploding loss when predicting close to zero, when used as an objective (loss) function, MAPE by its nature encourages the model to underestimate when predicting. The loss is also proportionately reduced when estimating large values, meaning that the punishment for large errors is the smallest when the electricity is the most expensive.

Instead, mean squared error (MSE) was used as the objective function during the training of these models. It is the most used regression loss function [20], with a smooth gradient fit for machine learning, eliminating the need for learning rate scheduling.


(2) $$\mathrm{MSE}(w,b)=\frac{1}{n}\sum_{i=1}^{n}{\left(y\left(x_{i}\right)-a_{i}\right)}^{2}$$

In addition to MSE and to better understand and compare the models, we measure and report the following metrics:

- MAPE measured in EUR or MW, described as above, with denominators between −1 and 1 being replaced by 1 to increase numerical stability.
- MAE (mean average error) measured in standard deviations, and EUR or MW.
- MAE represented as a percentage of the mean value (MAE%) to give another description of the model precision.

### 2.4. ANN Architectures Tested

The science behind ANNs constantly evolves, resulting in architectures better fit for the task. In this research, three main approaches were tested:

- A traditional neural network, with densely (fully) connected layers.
- A recurrent neural network (RNN) using LSTM cells, as a commonly used architecture for time series prediction, because of the ability of RNN to capture nonlinear short-term time dependencies [21].
- A temporal convolutional network (TCN or 1D CNN) [22], as a recent architecture showing promising performance in some time-series prediction tasks, such as weather pattern prediction [4].

Autoregressive models as well as a combination of these architectures were also created and tested.

#### 2.4.1. Densely Connected Layers

**Figure 4:**
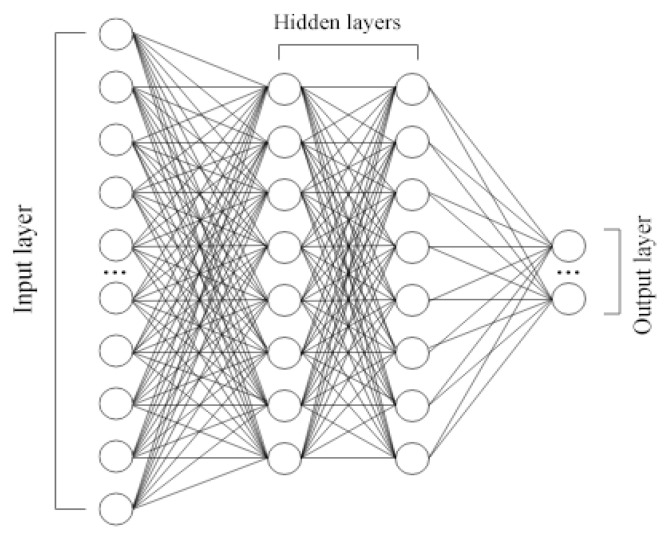
ANN with fully connected layers.

A traditional neural network architecture contains a hidden layer consisting of one or more columns of fully connected layers (Figure 4). A rectified linear unit (ReLU) was chosen as the activation function to optimize convergence and gradient preservation [23,24]. Testing the performance of a basic architecture and comparing it to linear regression and naive prediction can give us a better picture of the value that neural networks bring to the prediction.

Multiple configurations were tested with and without dropout layers to combat overfiting [25]. A single layer with 512 neurons (reported as “Dense” in results) and no dropout layers provided the best results for both the price and load prediction.

#### 2.4.2. Temporal Convolutional Layers

The ability of temporal convolutional networks to detect seasonal trends at different scales has made them a viable choice for certain time-series prediction tasks, such as short-term weather forecasting [8].

Two things specific to predicting hourly day-ahead electricity metrics influence the use of TCNs for the problem:

- The exact number of an hour in a day and a day in a week greatly influence the value to be predicted, which makes the pooling steps (such as max pooling), common to the convolutional networks, harder to model.
- On the other hand, a “similar day method”, looking through historical data to find the most similar day to today’s, to predict tomorrow as the day that followed that earlier day, has shown very good results in this field [1,5,6,26]. The pragmatic principle that guides this method underscores that the fact that two days are similar in this way convolutes many factors that contribute to this similarity but were not a part of the dataset.

The temporal convolutional network built for two problems tries to expand on the premise of the “similar day method” and use TCN’s feature extraction power to create a detection of “similar features” and combine them, potentially with help from another architecture, to give a better prediction.

**Figure 5:**
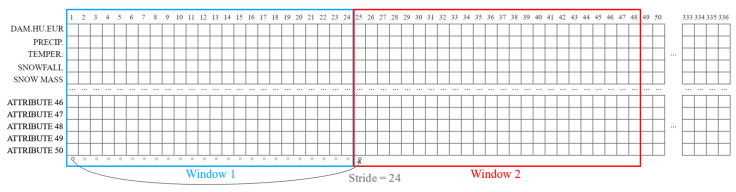
TCN windows for price prediction model.

To achieve entire day filtration, a filter width of 24 and a stride of 24 was chosen, to divide a set of 336 values into 15 windows of 24 values (Figure 5). A single convolutional layer of 256 filters (reported as “TCN” in results) showed the best performance.

In addition to this model, a model with said TCN layer and additional densely connected layer with 32 neurons (reported as “TCN_DENSE” in results) was created and tested, with the idea that the densely connected layer can provide additional conclusions from the features extracted by TCN layer.

#### 2.4.3. RNN Layer with LSTM Cells

Elman and Jordan’s architecture of recurrent neural networks, invented and used before LSTMs, was often efficient for time-series prediction tasks, including electricity price [27] and load [28] prediction. In comparison to “feed-forward” networks, these architectures were able to use the internal state (often called memory) to help process input sequences [29]. The recurrence of these networks means that calculations from the earlier step are passed to the next step, along with the input sequence.

In practice, recurrent networks were constrained with the need for careful balancing of the influence of previous calculations to future steps. This influence was shown to either vanish or become disproportionately large [3]. These two problems, known as vanishing and exploding gradient [30], led to RNN architectures often being unable to achieve the full promise of the idea behind them [31].

**Figure 6:**
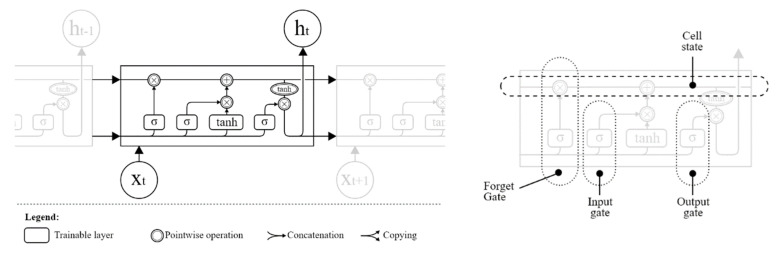
LSTM architecture and cell.

Long short-term memory cells (Figure 6) were one of the first implementations of RNNs to overcome this issue and enable networks to “forget the unimportant, and remember important information” [32].

Due to large LSTM layers overfitting quicker [15], somewhat simpler LSTM models have shown the best performance on the task and datasets used for this paper. A layer of 32 LSTM cells showed the best performance for both tasks.

In addition, a combined model (reported as Dense_LSTM_Dense) was created, where a hidden layer consists of an LSTM layer put between two fully connected layers, the first one having 128, and the second one 64 neurons, in order to help the recurrent network generalize better.

**Figure 7:**
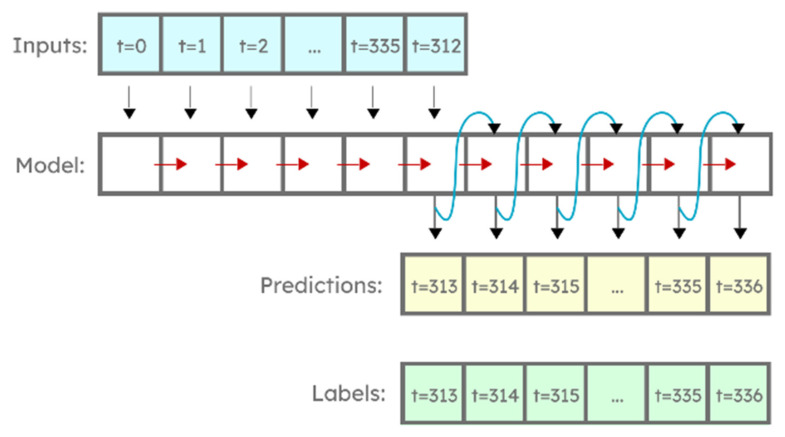
Autoregressive LSTM used in this test.

Moreover, an autoregressive LSTM model was created (reported as AR LSTM) that predicts only a single hour ahead and then feeds that value as an input to predict the next hour, repeating the step 24 times to get a full-day prediction (Figure 7).

### 2.5. Benchmark Values: Naive Model and Linear Regression

Two models that do not feature neural networks were created in order to evaluate the increase in precision when using neural networks: a naive one (reported as “Naive”) predicting tomorrow as a repeat of today, and a trainable linear regression model (reported as “Linear), using linear regression, effectively a single-neuron densely connected layer.

## 3. Results

### 3.1. Electricity Price Prediction

**Figure 8:**
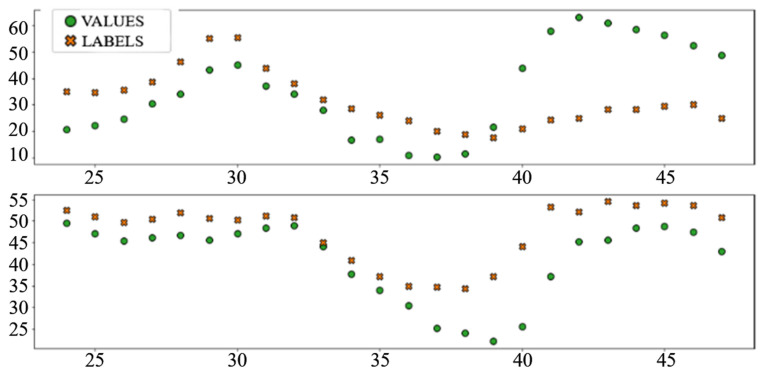
Price prediction: benchmark model “naïve”. Due to the nature of the prediction (only the last 24 h of the input window are considered) the prediction naïve for naive model are marked 24–47 on the *X*-axis, instead of 336–359.

**Figure 9:**
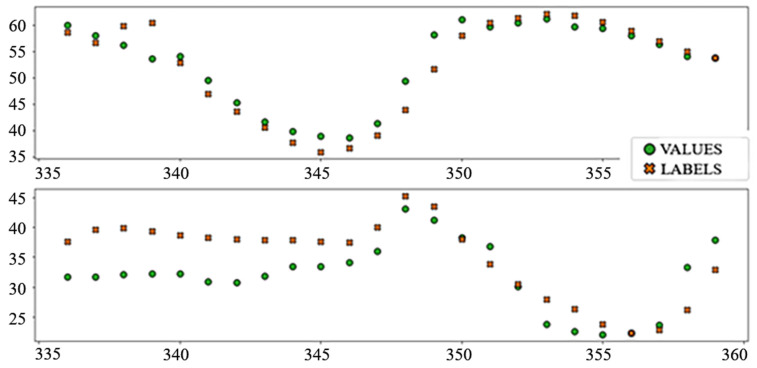
Price prediction: benchmark model “Linear”.

**Figure 10:**
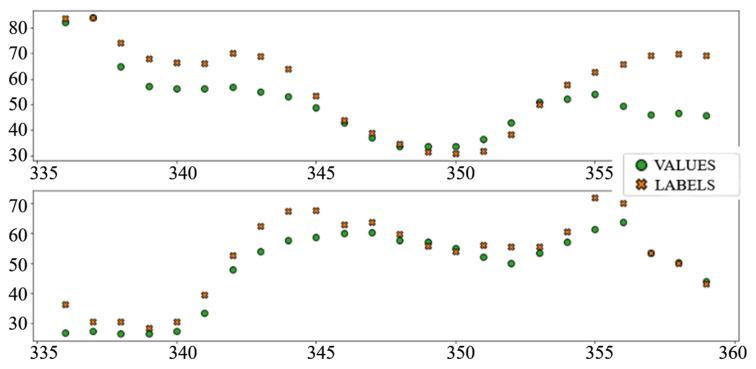
Price prediction examples: “Dense”.

**Figure 11:**
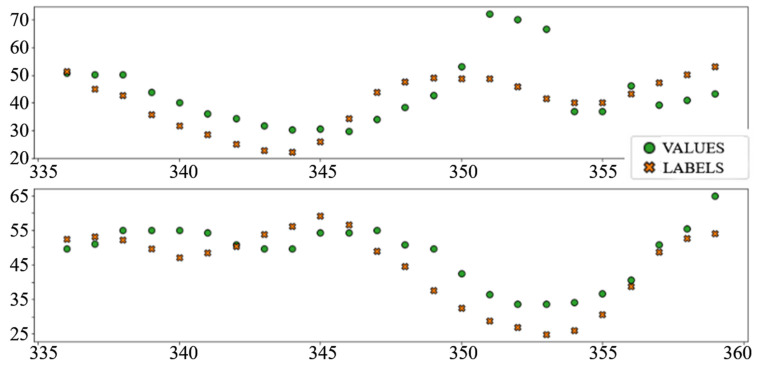
Price prediction examples: “TCN”.

**Figure 12:**
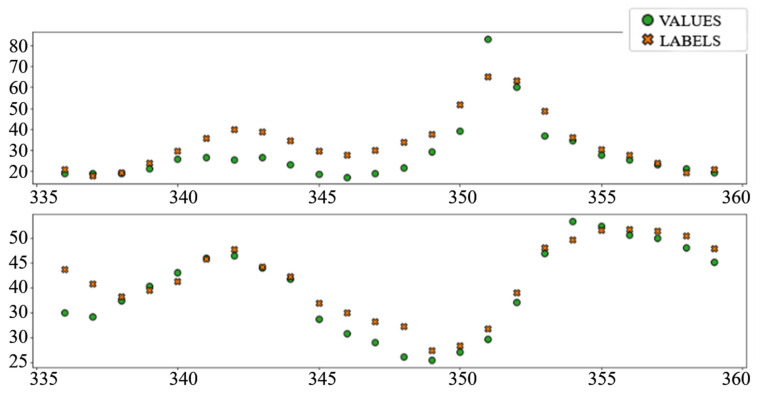
Price prediction examples: “TCN_Dense”.

**Figure 13:**
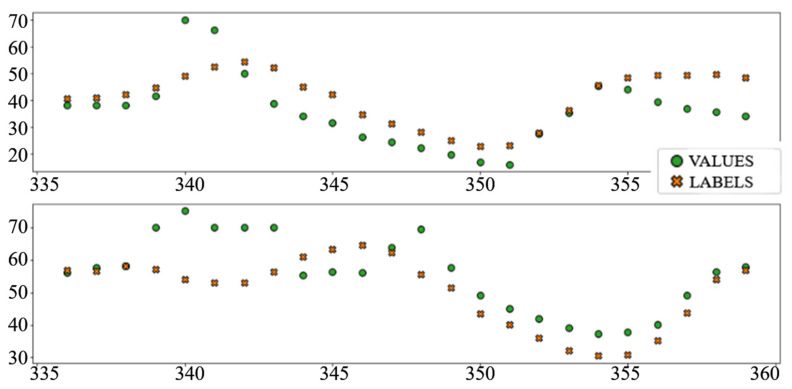
Price prediction examples: “LSTM”.

**Figure 14:**
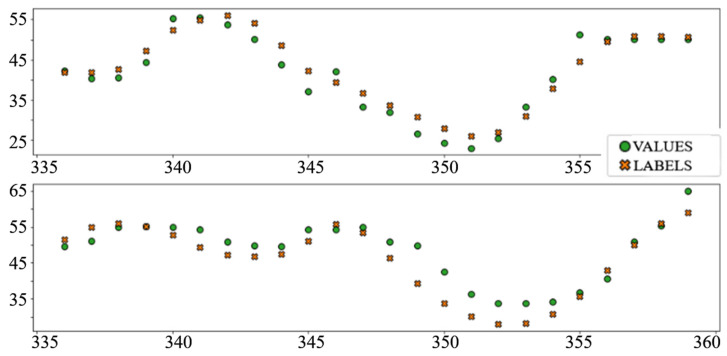
Price prediction examples: “Dense_LSTM_Dense”.

**Figure 15:**
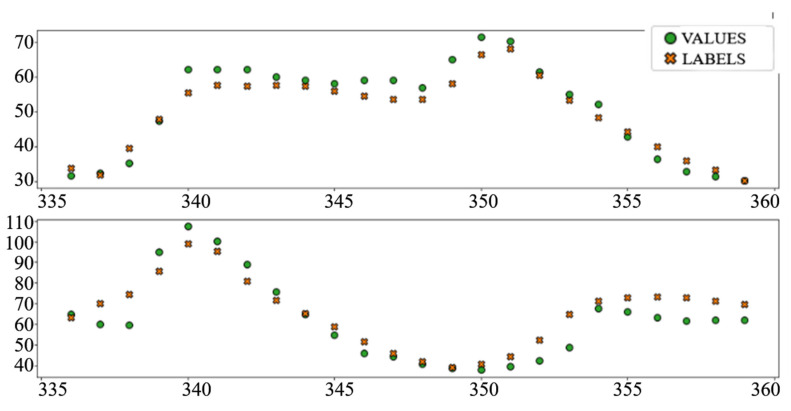
Price prediction examples: “AR LSTM”.

The best performing column in Table 3 is shaded. The following diagrams show example predictions (crosses) next to real values that had to be predicted (circles) for benchmark models (Figure 8 and Figure 9) and each of the ANN models (Figure 10, Figure 11, Figure 12, Figure 13, Figure 14 and Figure 15) for this dataset and task:

### 3.2. Electricity Load Prediction

Table 4 shows the performance of the created models for the problem of electricity load prediction. The final row contains the metrics of the official day-ahead predicted load, published on the ENTSO-E platform.

**Figure 16:**
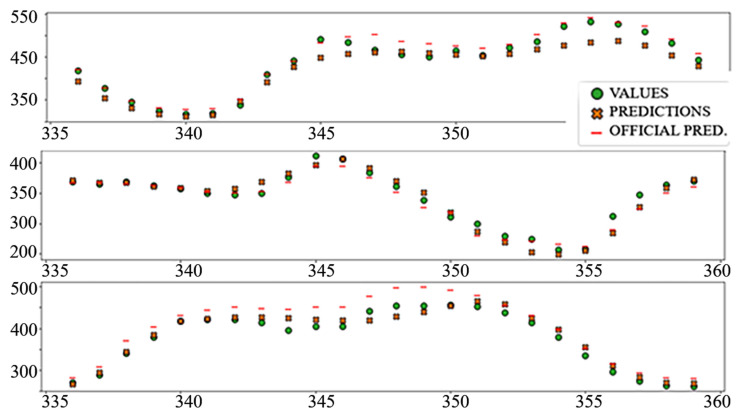
Load prediction: “Dense” model.

**Figure 17:**
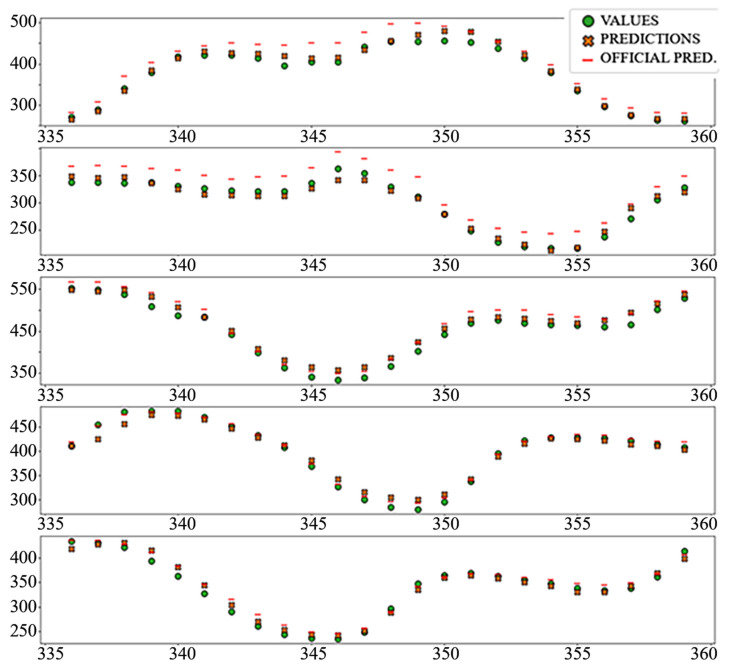
Load prediction: “TCN_Dense” model.

In the interest of clarity and space preservation, only the diagrams showing example predictions of the “Dense” (Figure 16, in order to show basic ANN performance for this task) and “TCN_dense” (Figure 17, to show the accuracy of the best performing model) are shown. In addition to markers shown in Figure 8 and Figure 9, an additional marker for the official prediction is added to each hour.

## 4. Discussion

Neural network models display significant improvement in accuracy compared to naive models, linear regression, and in the case of the consumption dataset, official prediction. Temporal convolutional layers and LSTM layers, both in combination with fully connected layers, demonstrated the best performance for both prediction tasks.

When comparing to the official prediction, it is worth noting that the accuracy of the official prediction for Montenegro has degraded in the part of the dataset from April 2021 onward. This led to the MAE of the official prediction on the test set being significantly larger than on the entire dataset (24.8 MW compared to 16.4 MW). Even in comparison to the entire dataset metrics (Table 5), the ANN models display significant improvement in terms of accuracy.

**Figure 18:**
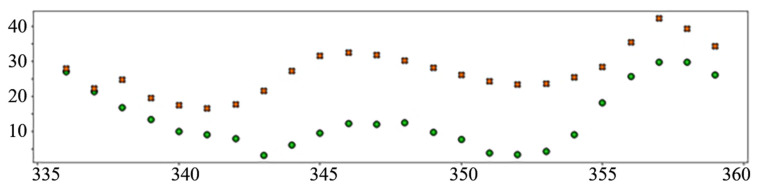
An example of a wrong AR LSTM prediction.

The relatively poor performance of the autoregressive model (AR LSTM), especially when predicting prices, is worth further exploration. In most cases, the model performs on par or better than other ANN prediction models, especially in later hours, but in cases where all models make large mistakes, AR LSTM performs even worse (Figure 18).

When working with more complex datasets that can reduce the frequency of these wrong predictions, the performance of an autoregressive model may become significantly better, and they might prove useful in ensemble models where a final prediction is derived from the prediction of several alternative models.

Temporal convolutional networks have shown very promising performance, implying their usability in the field of electricity day-ahead metric prediction. The 24-wide filter with a step of 24 was shown to be a robust feature-extraction tool in these networks and these layers should be considered, especially where datasets lack information and the “similar day” method has shown traditionally good results.

The performance of networks with fully connected layers is respectable, especially when having in mind their robustness, ease of implementation through many of the popular software packages, and a wide choice of ways to address issues like overfitting. This makes them a good solution for the quick creation of predictive models on similar datasets where more coarse prediction is acceptable.

Expanding the dataset with additional variables is a logical next step in achieving additional accuracy. More weather stations covering the entirety of the HUPX trading territory, preferably expanded with the wind data, is another way to increase the precision of the prediction. This additional data include, but are not limited to the following data that experts and current models already use:

- Consumption and production data for price prediction models [6]
- One-hot markers for holidays for price prediction models [6,33]
- Markers for the pre-holiday period [6]
- Separate supply and demand curves for the price prediction models [34]
- Weather forecast [33]
- Oil and gas price [19]
- Uranium and coal price [17]
- Dry and wet bulb temperature [35]

When using these models to create more complex and more accurate prediction models, we suggest considering these fields for future development:

- Creating more complete datasets, as stated above. When it comes to price prediction, it includes more complete weather data for countries participating in the market, weather forecast, supply and demand forecast, holiday and pre-holiday markers, and market prices of energy sources. When it comes to the consumption dataset, it mostly includes more complete weather data, weather forecasting, and better marking of pre-holidays and holidays.
- Exploring the potential increase in accuracy when using GRU (gated recurrent unit) instead of LSTM cells in RNN architectures. Research performed by Ugurlu and Oksuz [33] has shown the GRU achieving better accuracy when predicting electricity prices on the Turkish day-ahead market. Their experiments have shown that three-layer GRU structures display a statistically significant improvement in performance compared to other neural networks and statistical techniques for the Turkish market.
- Testing the effects of choosing Huber loss [20] as the objective function (instead of MSE) or introducing momentum or Nesterov momentum [36] into the gradient descent. Huber loss has been shown to be more robust than MSE when used on datasets with common anomalous spikes and dips, and the price dataset fits this description. Introducing momentum may increase training speed, which can be of great use in the future creation, testing, and comparison of algorithms.
- Re-training entire models on the unified training and validation set, or the entire set (training, validation, and test). This may lead to an increase in accuracy. Though not in line with the best practices of machine learning, training on the complete dataset may be of crucial importance to some time-series where the most recent data hide the most important conclusions [37]. The fact that user habits, regulation, and market conditions important for electricity trading often change may point to the most recent data in these especially important datasets, and training in this way significantly improves accuracy, though proving the existence of that improvement in the environment where test data were used for training becomes a problem of its own.
- Alternatively, instead of training on the entire dataset, roll-forward partitioning can be used [38] as an iterative method for training on almost the entire dataset while preserving the validation capabilities.

Algorithms for short-term electricity market price prediction are of use to all entities whose interest is connected to trading electricity and financially binding day-ahead prices, including:

- Participants in market trading, as a tool to help plan and (partially or fully) automate trading process [39].
- Reversible plant operators, to optimally plan the accumulation and production of electricity [40].
- Operators of hydro and other power plants with accumulation [41], as a tool to achieve optimal performance and maximize profit.
- Smart house owners, to plan and automate the optimal work of devices [42].
- Owners of electric cars and house batteries (such as Tesla PowerWall), in order to plan for accumulation when the electricity price is low and usage (or returning to the grid for profit) when the price is high [17].

Consumption (load) prediction models are widely used in electricity production and distribution systems, power plants, markets, building energy usage estimation, and other fields [43] and have additional use in some price prediction algorithms [34].

With each of the approaches to day-ahead electricity metric forecast having their own strengths and weaknesses, combining multiple approaches to give a more accurate prediction, and overcome weaknesses of a single model shows great promise. An ensemble (committee) model that combines multiple approaches is the most common way to combine predictions. Although some promising work has been undertaken in the field [44], major issues with comparing methods and predictions [6] are only amplified when it comes to combining them. Further improvement in this field which should result in a universal testing ground for electricity price forecasting algorithms should probably improve the research concerning ensemble algorithms.

With the increasing number of market and grid participants depending on these two prediction problems, and frequent changes of market conditions, the power of artificial neural networks is becoming an important part of these predictive processes. These results show their usefulness for the HUPX market and Montenegrin power consumption and reveal the promising usefulness of the temporal convolutional layers in these networks, especially when combined with other architectures.

With this work, we aimed to create a comparison of popular and promising ANN architectures for day-ahead electricity price forecasting on a common testing ground. We hope it will provide a valuable insight for researchers aiming to create, improve, and combine algorithms and methods for short-term electricity metric prediction, and encourage them to consider the promise of artificial neural networks when approaching this largely unexplored topic.

## Figures and Tables

**Table 1:** HUPX dataset descriptive statistics.

	Mean	STD	Min	50%	Max
Price (EUR)	46.408	20.453	−113.67	44.59	300.1
Precipitation (mm)	0.0726	0.1792	0	0.006	4.03
Temperature (C)	11.528	10.0697	−16.777	11.426	39.128
Snowfall (cm)	0.0065	0.0388	0	0	1.5869
Snow mass (cm)	0.9443	2.6921	0	0	25.074
Cloud cover %	0.5228	0.3223	0	0.541	0.9988
Air density (kg/m^3^)	1.2176	0.045	1.1108	1.2137	1.3662

**Table 2:** Montenegrin power consumption (load) descriptive statistics, ENTSO-e transparency.

	Mean	STD	Min	50%	Max
Actual Load	378.17	77.69	0.0	384.0	838
Forecasted Load	386.32	78.06	0.0	373.0	629

**Table 3:** Performance of models for electricity price prediction.

Model	MSE (Std)	MAE (EUR)	MAE (Std)	MAPE	MAE (%)
naïve	1.6676	18.03013	0.8815	50.0480	38.85116
Linear	0.2688	7.74385	0.3786	21.2923	16.68639
Dense	0.2199	6.96251	0.3404	20.1414	15.00276
TCN	0.2186	7.10978	0.3476	20.6052	15.32010
TCN_dense	0.2115	6.83161	0.3340	19.450	14.72069
LSTM	0.2390	7.06683	0.3455	19.9769	15.22754
Dense_LSTM_dense	0.2177	6.70070	0.3276	19.5300	14.43862
AR LSTM	0.2423	7.21818	0.3529	21.2600	15.55369

**Table 4:** Performance of models for electricity load prediction in comparison to the official prediction.

Model	MSE (Std)	MAE (Std)	MAE (MW)	MAPE	MAE(%)	MAE% Δ Off.
Naive	2.0300	1.0459	81.1417	24.3706	21.4539	14.8974
Linear	0.1344	0.2813	21.8234	6.1125	5.7699	−0.7859
Dense	0.1017	0.2472	19.1779	5.5690	5.0705	−1.4854
TCN	**0.0628**	0.1921	14.9032	4.2585	3.9403	−2.6156
TCN_dense	0.0648	**0.1906**	**14.7869**	**4.1269**	**3.9095**	**−2.6463**
LSTM	0.0684	0.1989	15.4308	4.4730	4.0798	−2.4761
Dense_LSTM_dense	0.0651	0.1927	14.9498	4.2285	3.9526	−2.6033
AR LSTM	0.0789	0.2198	17.0522	5.01	4.5085	−2.0474
**Official**	**0.1617**	**0.3196**	**24.7961**	**7.8306**	**6.5559**	/

**Table 5:** Official load prediction on full and test dataset.

Official Prediction	MSE (Std)	MAE (Std)	MAE (MW)	MAPE	MAE%
Full dataset	0.0834	0.2114	16.4026	4.7091	4.3367
Test dataset	0.1617	0.3196	24.7961	7.8306	6.5559

## Data Availability

HUPX historical day-ahead market hourly prices acquired from the datasets found on Hungarian Power Exchange website: https://hupx.hu/en/market-data/dam/historical-data (accessed on 19 May 2021). Weather data for Hungary acquired from Renewables.ninja Weather (hourly data, 1980–2019)—Version: 1.3, https://www.renewables.ninja/country_downloads/HU/ninja_weather_country_HU_merra-2_land_area_weighted.csv (accessed on 18 May 2021). Consumption/load data acquired and assembled from the European Network of Transmission System Operators for Electricity (ENTSO-E) transparency platform, https://transparency.entsoe.eu/load-domain/r2/totalLoadR2/show (accessed on 21 June 2021).

## References

[1] Shahidehpour M., Yamin H., Li Z. (2003). Market Operations in Electric Power Systems: Forecasting, Scheduling, and Risk Management.

[2] Pavićević M., Popović T. Forecasting Day-Ahead Electricity Price with Artificial Neural Networks: A Comparison of Architectures. Proceedings of the 11th IEEE International Conference on Intelligent Data Acquisition and Advanced Computing Systems: Technology and Applications (IDAACS).

[3] Graves A., Liwicki M., Fernández S., Bertolami R., Bunke H., Schmidhuber J. (2009). A Novel Connectionist System for Unconstrained Handwriting Recognition. IEEE Trans. Pattern Anal. Mach. Intell..

[4] Yan J., Mu L., Wang L., Ranjan R., Zomaya A.Y. (2020). Temporal Convolutional Networks for the Advance Prediction of ENSO. Sci. Rep..

[5] Weron R. (2007). Modeling and Forecasting Electricity Loads and Prices: A Statistical Approach.

[6] Weron R. (2014). Electricity price forecasting: A review of the state-of-the-art with a look into the future. Int. J. Forecast..

[7] Aggarwal S.K., Saini L.M., Kumar A. (2009). Short term price forecasting in deregulated electricity markets: A review of statistical models and key issues. Int. J. Energy Sect. Manag. IJESM.

[8] Hewage P., Behera A., Trovati M., Pereira E., Ghahremani M., Palmieri F., Liu Y. (2020). Temporal convolutional neural (TCN) network for an effective weather forecasting using time-series data from the local weather station. Soft Comput..

[9] Hungarian Power Exchange. https://hupx.hu/en/#.

[10] ENTSO-E Transparency Platform. https://transparency.entsoe.eu/.

[11] Keras: The Python Deep Learning API. https://keras.io/.

[12] TensorFlow. https://www.tensorflow.org/.

[13] Pandas—Python Data Analysis Library. https://pandas.pydata.org/.

[14] NumPy. https://numpy.org/.

[15] Brownlee J. (2018). Deep Learning for Time Series Forecasting—Predict the Future with MLPs, CNNs and LSTMs in Python; 1.3.

[16] Nielsen M.A. (2015). Neural Networks and Deep Learning.

[17] Bouley C. (2020). Recurrent Neural Networks for Electricity Price Prediction, Time-Series Analysis with Exogenous Variables.

[18] Klešić I. (2018). Prognoza Cijene Električne Energije.

[19] Wagner A., Schnürch S. (2020). Electricity Price Forecasting with Neural Networks on EPEX Order Books. Appl. Math. Financ..

[20] Grover P. 5 Regression Loss Functions All Machine Learners Should Know. https://heartbeat.fritz.ai/5-regression-loss-functions-all-machine-learners-should-know-4fb140e9d4b0.

[21] Lindemann B., Müller T., Vietz H., Jazdi N., Weyrich M. (2021). A survey on long short-term memory networks for time series prediction. Procedia CIRP.

[22] Bai S., Kolter J.Z., Koltun V. (2018). An Empirical Evaluation of Generic Convolutional and Recurrent Networks for Sequence Modeling. arXiv.

[23] Brownlee J. A Gentle Introduction to the Rectified Linear Unit (ReLU). https://machinelearningmastery.com/rectified-linear-activation-function-for-deep-learning-neural-networks/.

[24] Pedamonti D. (2018). Comparison of non-linear activation functions for deep neural networks on MNIST classification task. arXiv.

[25] Srivastava N., Hinton G., Krizhevsky A., Sutskever I., Salakhutdinov R. (2014). Dropout: A Simple Way to Prevent Neural Networks from Overfitting. J. Mach. Learn. Res..

[26] Bierbrauer M., Menn C., Rachev S.T., Trück S. (2007). Spot and derivative pricing in the EEX power market. J. Bank. Financ..

[27] Beigaitė R., Krilavičius T. (2017). Electricity Price Forecasting for Nord Pool Data.

[28] Ruiz L.G.B., Rueda R., Cuéllar M.P., Pegalajar M.C. (2018). Energy consumption forecasting based on Elman neural networks with evolutive optimization. Expert Syst. Appl..

[29] Abiodun O.I., Jantan A., Omolara A.E., Dada K.V., Mohamed N.A., Arshad H. (2018). State-of-the-art in artificial neural network applications: A survey. Heliyon.

[30] Brownlee J. (2017). A Gentle Introduction to Long Short-Term Memory Networks by the Experts.

[31] Gers F.A., Schmidhuber J., Cummins F. (2000). Learning to Forget: Continual Prediction with LSTM. Neural Comput..

[32] Olah C. Understanding LSTM Networks. https://colah.github.io/posts/2015-08-Understanding-LSTMs/.

[33] Ugurlu U., Oksuz I. (2018). Electricity Price Forecasting Using Recurrent Neural Networks. Energies.

[34] Ziel F., Steinert R. (2016). Electricity price forecasting using sale and purchase curves: The X-Model. Energy Econ..

[35] Nateghi S.M.R. (2019). A Data-Driven Approach to Assessing Supply Inadequacy Risks Due to Climate-Induced Shifts in Electricity Demand. Risk Anal..

[36] Sutskever I., Martens J., Dahl G., Hinton G. On the Importance of Initialization and Momentum in Deep Learning. Proceedings of the 30th International Conference on Machine Learning, PMLR 28(3).

[37] Géron A. (2019). Hands-On Machine Learning with Scikit-Learn, Keras, and TensorFlow.

[38] Cochrane C. Time Series Nested Cross-Validation. https://towardsdatascience.com/time-series-nested-cross-validation-76adba623eb9.

[39] Bayram I.S., Shakir M.Z., Abdallah M., Qaraqe K. A Survey on Energy Trading in Smart Grid. Proceedings of the 2014 IEEE Global Conference on Signal and Information Processing (GlobalSIP).

[40] Čabarkapa R., Komatina D., Ćirović G., Petrović D., Vulić M. (2014). Analysis of Day-Ahead Electricity Price Fluctuations in the Regional Market and the Perspectives of the Pumped Storage Hydro Power Plant “bistrica”.

[41] Dunn R.I., Hearps P.J., Wright M.N. (2012). Molten-salt power towers: Newly commercial concentrating solar storage. Proc. IEEE.

[42] Morrison Sara Texas Heat Wave Overloads Power Grid, Causing Companies to Adjust Thermostats Remotely—Vox. https://www.vox.com/recode/22543678/smart-thermostat-air-conditioner-texas-heatwave.

[43] Danandeh Mehr A., Bagheri F., Safari M.J.S. (2020). Electrical energy demand prediction: A comparison between genetic programming and decision tree. Gazi Univ. J. Sci..

[44] Guo J.J., Luh P.B. (2004). Improving market clearing price prediction by using a committee machine of neural networks. IEEE Trans. Power Syst..

